# Phase II/III weekly *nab*-paclitaxel plus gemcitabine or carboplatin versus gemcitabine/carboplatin as first-line treatment of patients with metastatic triple-negative breast cancer (the tnAcity study): study protocol for a randomized controlled trial

**DOI:** 10.1186/s13063-015-1101-7

**Published:** 2015-12-16

**Authors:** Denise A. Yardley, Adam Brufsky, Robert E. Coleman, Pierfranco F. Conte, Javier Cortes, Stefan Glück, Jean-Mark A. Nabholtz, Joyce O’Shaughnessy, Robert M. Beck, Amy Ko, Markus F. Renschler, Debora Barton, Nadia Harbeck

**Affiliations:** Sarah Cannon Research Institute and the Tennessee Oncology, PLLC, 250 25th Avenue North, Suite 100, Nashville, TN 37203 USA; University of Pittsburgh Medical Center, Pittsburgh, PA USA; Weston Park Hospital, Sheffield Cancer Research Center, Sheffield, England; Department of Surgery, Oncology and Gastroenterology, University of Padova, Padova, and Istituto Oncologico Veneto IRCCS, Padova, Italy; Vall d’Hebron Institute of Oncology (VHIO), Barcelona, Spain; Celgene Corporation, Summit, NJ USA; Centre de Lutte Contre le Cancer d’Auvergne, Clermont Ferrand, France; Texas Oncology-Baylor Charles A. Sammons Center; US Oncology, Dallas, TX USA; Breast Center, University of Munich, Munich, Germany

**Keywords:** tnAcity, Triple-negative breast cancer, Metastatic, *Nab*-paclitaxel, Abraxane, Gemcitabine, Carboplatin, Pick-the-winner trial design

## Abstract

**Background:**

Triple-negative breast cancer is an aggressive disease with unmet clinical needs. In a phase III study of patients with metastatic triple-negative breast cancer, first-line gemcitabine/carboplatin resulted in a median progression-free survival of 4.6 months. *nab*-paclitaxel-based regimens (with gemcitabine or carboplatin ± bevacizumab) also demonstrated efficacy and safety in first-line phase II trials of human epidermal growth factor receptor 2-negative metastatic breast cancer.

**Trial design:**

In this international, multicenter, open-label, randomized phase II/III trial, the efficacy and safety of first-line *nab*-paclitaxel with gemcitabine or with carboplatin will be compared with gemcitabine/carboplatin (control arm) for metastatic triple-negative breast cancer.

**Methods:**

In the phase II portion, 240 patients with measurable metastatic triple-negative breast cancer and treatment-naive for metastatic disease will be randomized 1:1:1 (stratified by disease-free interval: ≤ 1 versus > 1 year) to *nab*-paclitaxel 125 mg/m^2^ plus gemcitabine 1000 mg/m^2^, *nab*-paclitaxel 125 mg/m^2^ plus carboplatin area under the curve 2 mg × min/mL, or gemcitabine 1000 mg/m^2^ plus carboplatin area under the curve 2 mg × min/mL, all given on days 1 and 8 of a 21-day cycle. Investigator-assessed progression-free survival (primary endpoint), overall response rate, overall survival, and safety will be assessed. A ranking algorithm of five efficacy and safety parameters will be used to pick the “winner” of the *nab*-paclitaxel regimens. In the phase III portion, 550 patients will be randomized 1:1 (stratified by disease-free interval: ≤ 1 versus > 1 year, and prior adjuvant/neoadjuvant taxane use) to the *nab*-paclitaxel combination arm selected from the phase II portion or to the control arm. Patients in phase II will not be part of the phase III population. The phase III primary endpoint is blinded, independently-assessed progression-free survival; secondary endpoints include blinded, independently-assessed overall response rate, overall survival, disease control rate, duration of response, and safety. Biomarker and circulating tumor-cell exploratory analyses and quality-of-life assessments will also be performed. A list of approving ethical bodies was provided in Additional file 1.

**Discussion:**

The tnAcity trial aims to identify a new standard cytotoxic chemotherapy regimen for first-line treatment of metastatic triple-negative breast cancer.

**Trial registration:**

ClinicalTrials.gov: NCT01881230. Date of registration: 17 June 2013.

**Electronic supplementary material:**

The online version of this article (doi:10.1186/s13063-015-1101-7) contains supplementary material, which is available to authorized users.

## Background

Triple-negative breast cancer (TNBC) accounts for approximately 15 to 20 % of all breast cancers and is associated with an aggressive clinical course and a poor prognosis [1–5]. TNBC tumors comprise a very heterogeneous group of cancers that are poorly differentiated and demonstrate high rates of proliferation; patients with TNBC face early disease recurrence and low overall survival rates compared with patients with other types of breast cancers. Furthermore, patients with recurring and/or metastatic TNBC (mTNBC) are more likely to develop visceral and brain metastases and generally have a shorter survival than patients with other breast cancer subtypes [1, 3, 6].

While TNBC represents a highly diverse group of cancers, it can be characterized by a lack of the estrogen receptor (ER) and progesterone receptor (PR) and by the absence of overexpression of the human epidermal growth factor receptor 2 (HER2) [4]. The genetic and molecular heterogeneity of these tumors, shorter median time from relapse to death, and lack of currently identified treatment targets make management of TNBC particularly challenging [2, 4]. Currently in the United States, the standard treatment option for patients with mTNBC outside of clinical trials is cytotoxic chemotherapy; however, these patients respond better to chemotherapy than do patients with breast cancers that are hormone-receptor-positive [7]. Adding a taxane to chemotherapy appears to improve outcomes in patients with TNBC [2]. In addition, platinum-containing regimens have demonstrated antitumor activity in this patient population [8, 9]. However, given the lack of prospective randomized data, no optimal standard-of-care regimen for patients with mTNBC currently exists. Treatment strategies for mTNBC are largely based on the National Comprehensive Cancer Network recommendations and other international guidelines for the overall population of patients with metastatic breast cancer [8, 10–12].

While monotherapy with sequential single-agent chemotherapy is preferred in most cases of metastatic breast cancer, the National Comprehensive Cancer Network guidelines also recommend some combination regimens that have been explored in mTNBC [8, 9, 13, 14]. Capecitabine plus docetaxel and gemcitabine plus solvent-based (sb-) paclitaxel treatment has demonstrated efficacy in patients with mTNBC [13, 14]. Another small, randomized phase II study in the first-line treatment of 53 patients with mTNBC, combination therapy with docetaxel plus capecitabine compared with docetaxel plus cisplatin suggests that the platinum-taxane-based regimen may be more effective than the capecitabine-based regimen based on overall response rate (ORR), progression-free survival (PFS), and overall survival (OS) [13].

The rationale for including platinum agents in the treatment of TNBC is supported by a growing body of preclinical and clinical evidence, with its basis in observed platinum-conferred sensitivity as a function of the impaired homologous recombination repair mechanisms present in TNBC and breast cancer characterized by mutations in the breast cancer susceptibility gene (*BRCA*) [15–18]. Increased sensitivity to cisplatin was demonstrated in a xenograft model of TNBC with *BRCA1*-deficient cells [19]. The recent phase III TNT trial evaluated carboplatin versus docetaxel treatment in patients with metastatic or recurrent locally advanced triple-negative or *BRCA1/2* mutated breast cancer [20]. Although results did not support superior activity of carboplatin compared with docetaxel in unselected patients with TNBC, treatment with carboplatin resulted in a significantly higher ORR versus docetaxel in patients with *BRCA1/2* mutations (68 % versus 33.3 %; *P* = 0.03). However, in the multicenter, single-arm phase II TBCRC009 trial evaluating single-agent platinum agents in mTNBC, 6 patients (7 %) who achieved durable responses (34–69 months) were long-term survivors and remained off all therapy; all of these patients had *BRCA1/2* wild type (5) or unknown (1) mutation status and had received platinum therapy as first-line or second-line therapy for mTNBC [17].

The TBCRC009 trial also evaluated first-line or second-line treatment with cisplatin or carboplatin in patients with mTNBC [17]. Grouped efficacy results included an ORR of 26 % and a median PFS of 2.9 months (*n* = 86), with a higher ORR in patients receiving first-line treatment than in those receiving treatment in the second-line setting (29 % versus 12 %).

Platinum agents in chemotherapy combinations have demonstrated activity in patients with TNBC both in the neoadjuvant and metastatic settings [9, 16, 21, 22]. In the metastatic setting, a randomized phase II study compared the addition of iniparib with gemcitabine plus carboplatin in patients with TNBC. Gemcitabine plus carboplatin resulted in a median PFS of 3.6 months and a median OS of 7.7 months (*n* = 62) [16]. In a subsequent confirmatory randomized phase III study comparing these 2 regimens, gemcitabine plus carboplatin demonstrated median PFS and OS of, respectively, 4.1 and 11.1 months in the intent-to-treat (ITT) population and 4.6 and 12.4 months based on an exploratory analysis in patients receiving first-line treatment [9]. Even though the phase III trial did not meet its prespecified criteria for the coprimary endpoints of PFS (*P* = 0.027) and OS (*P* = 0.28), it provided a baseline for sample size calculation for future randomized trials in mTNBC.

*nab*-paclitaxel, an albumin-bound, 130-nm-particle formulation of paclitaxel (Abraxane; Celgene Corporation, Summit, NJ, USA), has demonstrated antitumor activity and a favorable safety profile in patients with HER2-negative metastatic breast cancer, including those with mTNBC [23–25]. *nab*-paclitaxel was developed to improve the efficacy of taxane treatment while reducing the toxicity typically associated with the solvents used to formulate paclitaxel and docetaxel [26–28]. In a pivotal phase III trial, *nab*-paclitaxel demonstrated a significantly higher ORR, significantly longer time to progression, and significantly greater OS in patients with metastatic breast cancer receiving second-line or higher treatment compared with sb-paclitaxel [26]. Based on these findings, *nab*-paclitaxel was approved by the US Food and Drug Administration in 2005 for the treatment of patients with metastatic breast cancer after failure of combination chemotherapy for metastatic disease or relapse within 6 months of adjuvant chemotherapy. Prior therapy should have included an anthracycline unless clinically contraindicated [29]. A subsequent phase II trial in patients with metastatic breast cancer demonstrated that *nab*-paclitaxel administered the first 3 of 4 weeks (qw 3/4) was a feasible and effective regimen [27, 28]. Recently *nab*-paclitaxel has also demonstrated efficacy in patients with TNBC in the neoadjuvant treatment setting [30]. In the large phase III GeparSepto trial, patients with early breast cancer received either weekly *nab*-paclitaxel 125 mg/m^2^ (reduced from 150 mg/m^2^) or sb-paclitaxel, each followed by epirubicin and cyclophosphamide. Treatment with *nab*-paclitaxel resulted in a significantly higher pathological complete response (pCR) rate than sb-paclitaxel (38 % versus 29 %; *P* = 0.001), and this effect was also seen in the subgroup of patients with TNBC (*n* = 275; 48.2 % versus 25.7 %; *P* < 0.001).

The addition of *nab*-paclitaxel to platinum-based therapy has been shown to be an active regimen in patients with mTNBC [25]. A phase II trial of *nab*-paclitaxel 100 mg/m^2^ qw 3/4 in combination with carboplatin area under the curve (AUC) 2 qw 3/4 and bevacizumab 10 mg/kg every 2 weeks (q2w) demonstrated promising antitumor activity in patients with mTNBC, resulting in an ORR of 85 %, a median PFS of 9.2 months, and an acceptable safety profile [25]. A phase II study evaluating the combination of *nab*-paclitaxel plus carboplatin as first-line treatment for mTNBC is currently under way [31]. Weekly *nab*-paclitaxel plus carboplatin has also demonstrated robust antitumor activity and an acceptable tolerability profile in the setting of advanced non-small cell lung cancer (NSCLC) [32]. In a phase III trial of patients with advanced NSCLC, weekly *nab*-paclitaxel plus carboplatin significantly improved the ORR (the primary endpoint) compared with sb-paclitaxel.

The combination of *nab*-paclitaxel plus gemcitabine has been shown to be effective and tolerable in a phase II trial in patients with previously untreated metastatic breast cancer (*n* = 50), including those with TNBC [23]. Treatment with *nab*-paclitaxel 125 mg/m^2^ plus gemcitabine 1000 mg/m^2^ on days 1 and 8 of a 3-week cycle resulted in an ORR of 50 % and a median PFS of 7.9 months. Furthermore, in the subset of patients with mTNBC (*n* = 13), the *nab*-paclitaxel combination resulted in an ORR of 77 %. In another phase II trial (*n* = 30), the addition of bevacizumab 10 mg/kg q2w to *nab*-paclitaxel 150 mg/m^2^ plus gemcitabine 1500 mg/m^2^ resulted in an ORR of 75.9 % and a median PFS of 10.4 months in patients with metastatic breast cancer [24]. As in the aforementioned trial, this *nab*-paclitaxel-based combination in patients with TNBC (*n* = 13) demonstrated similar activity, with an ORR of 69 %. More recently, the combination of *nab*-paclitaxel and gemcitabine has been approved and is now standard therapy for patients with metastatic pancreatic cancer based on a randomized phase III trial of *nab*-paclitaxel and gemcitabine versus gemcitabine (MPACT). The study demonstrated a significant improvement in overall survival, response rate, and PFS, and the combination was well-tolerated [33, 34]. Taken together, these findings support further evaluation of this combination in the mTNBC setting.

Here, we present the study design of the phase II/III tnAcity (Triple-Negative Albumin-bound paclitaxel Combination International Treatment Study) trial, which will evaluate the efficacy and safety of two *nab*-paclitaxel combination regimens (with either gemcitabine or carboplatin) compared with that of gemcitabine plus carboplatin as treatment for mTNBC. The dosages and schedule of administration of gemcitabine and carboplatin were adjusted to be the same across treatment arms.

## Methods/Design

### Overall study design

**Fig. 1:**
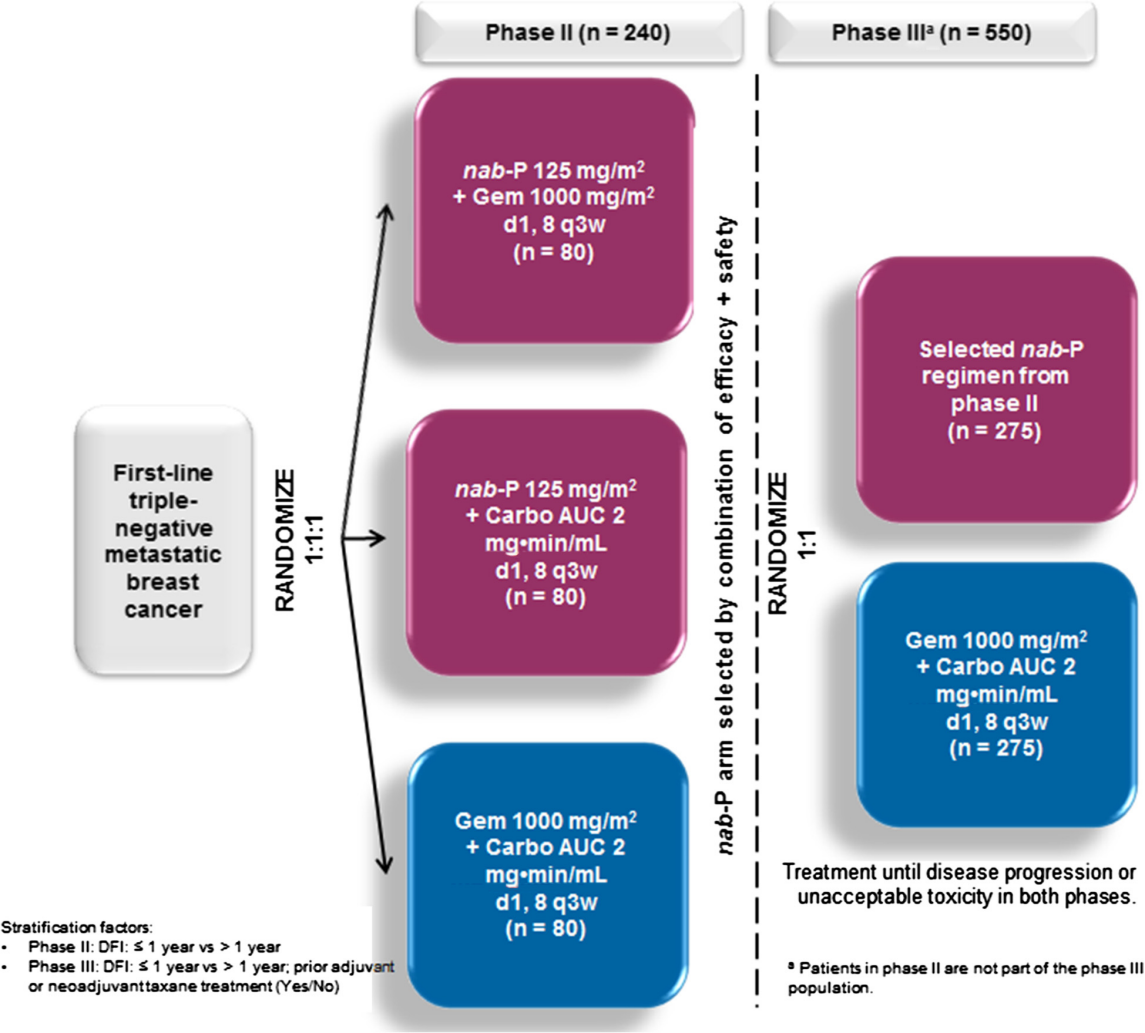
Overall study design. AUC, area under the curve; Carbo, carboplatin; d, day; DFI, disease-free interval; Gem, gemcitabine; *nab*-P, *nab*-paclitaxel; q3w, every 3 weeks

tnAcity is an international, multicenter, open-label, randomized phase II/III trial designed to test weekly *nab*-paclitaxel in combination with gemcitabine or carboplatin, compared with gemcitabine plus carboplatin, as first-line treatment for patients with mTNBC. Patients will be enrolled at approximately 150 sites globally. The aim of the randomized phase II portion of the study is to assess the safety and efficacy of *nab*-paclitaxel plus carboplatin and *nab*-paclitaxel plus gemcitabine in a homogeneous mTNBC patient population. A defined ranking algorithm will then determine the selection of one of these *nab*-paclitaxel-based regimens as the experimental arm to be compared with gemcitabine plus carboplatin in the phase III portion of the study (Fig. 1).

### Participants

A total of 790 women (approximately 240 patients in the phase II portion and 550 patients in the phase III portion) who are at least 18 years old with measurable mTNBC (ER-negative, PR-negative, and lacking HER2 overexpression) will be enrolled in this study. No prior cytotoxic chemotherapy for metastatic disease is allowed, but patients may have received prior adjuvant or neoadjuvant therapy. Patients must have previously received adjuvant or neoadjuvant anthracycline therapy, unless not indicated by the treating physician. Patients in the phase II portion of the study will not be part of the phase III population. Table 1 summarizes the major eligibility criteria.

**Table 1:** Eligibility criteria for the Triple-Negative Albumin-bound paclitaxel Combination International Treatment Study (tnAcity) trial

Key inclusion criteria	Key exclusion criteria
Female, aged ≥ 18 years	Concurrent chemotherapy or any other antitumor breast cancer therapy
Measurable metastatic disease	
Pathologically confirmed as triple-negative, defined as ER and PR expression both < 1 % of tumor cell nuclei per ASCO/CAP guidelines^a^ and HER2 negative per ASCO/CAP guidelines^a^ (IHC 0 or 1+ or FISH−, or IHC 2+ and FISH−)	Concurrent chemotherapy or any other antitumor therapy for breast cancer. Prior immunotherapy or monoclonal antibody therapy for metastatic breast cancer is acceptable
If prior ER/PR/HER2+ breast cancer history, must have pathologically confirmed TN disease in ≥ 1 metastatic site	Receipt of prior cytotoxic chemotherapy after incomplete resection of locoregional recurrent disease
ECOG PS 0–1	History or current evidence of brain metastasis, including leptomeningeal involvement
No prior cytotoxic chemotherapy for metastatic disease; prior radiation therapy allowed	History of other primary malignancy in the past 5 years, except prior history of breast or in situ/basal/localized squamous cell skin cancer
Must have received previous adjuvant or neoadjuvant anthracycline therapy unless not indicated by physician	Baseline peripheral neuropathy grade ≥ 2 by NCI CTCAE v4.0
Patients with newly diagnosed mTNBC are eligible if anthracycline not indicated by physician	Patients with bone as the only site of metastatic disease
Completion of prior neoadjuvant or adjuvant chemotherapy ≥ 6 months before randomization or ≥ 12 months if containing taxane, gemcitabine, or platinum agents	Patients with regional lymph node as the only site of metastatic disease

^a^Local pathology review will be conducted following ASCO CAP guidelines (2013 preferred, 2007 acceptable)

*ASCO*, American Society of Clinical Oncology, *CAP* College of American Pathologists, *ECOG PS* Eastern Cooperative Oncology Group performance status, *ER* estrogen receptor, *FISH* fluorescence in situ hybridization, *HER2* human epidermal growth factor receptor 2, *IHC* immunohistochemistry, *mTNBC* metastatic triple-negative breast cancer, *NCI CTCAE* National Cancer Institute Common Terminology Criteria for Adverse Events, *PR* progesterone receptor, *TN* triple-negative, *TNBC* triple-negative breast cancer

### Ethical considerations and approvals

This study is being conducted with Good Clinical Practice as described in the International Conference on Harmonization Guideline E6 and in accordance with general ethical principles outlined in the Declaration of Helsinki. All relevant ethical approvals from institutional review board/independent ethics committee have been obtained prior to commencement (see Additional file 1 for details). Investigators will act in accordance with applicable national and local laws of pertinent regulatory authorities. The trial is registered at ClinicalTrials.gov (NCT01881230).

### Consent

Consent must be obtained by the investigator prior to any study-related procedures. If a protocol is amended and it impacts the content of the informed consent, the consent document must be revised.

### Therapy response assessment

Response will be assessed by Response Evaluation Criteria In Solid Tumors (RECIST) 1.1 guidelines on images obtained with computed tomography scans. Scans will be performed at screening and every 6 weeks (±5 days) from randomization. Safety will be evaluated according to the National Cancer Institute Common Terminology Criteria for Adverse Events (NCI CTCAE) Version 4.0.

### Intervention and outcomes

#### Phase II portion of the study

The primary objective of the open-label phase II portion of this trial is to evaluate the efficacy and risk/benefit profiles of the two *nab*-paclitaxel experimental treatments in order to identify the *nab*-paclitaxel combination that will be used in the phase III portion of the study (selection algorithm discussed later). Approximately 240 patients with mTNBC and no prior chemotherapy for metastatic disease (*n* = 80 per arm) will be randomized 1:1:1, stratified by disease-free interval (DFI; ≤ 1 year versus > 1 year), to 2 *nab*-paclitaxel combination arms and 1 comparator arm: arm A (*nab*-paclitaxel 125 mg/m^2^ plus gemcitabine 1000 mg/m^2^), arm B (*nab*-paclitaxel 125 mg/m^2^ plus carboplatin AUC 2 mg × min/mL), and arm C (gemcitabine 1000 mg/m^2^ plus carboplatin AUC 2 mg × min/mL) given intravenously (IV) on days 1 and 8 of a 21-day cycle. Enrollment will continue until at least 240 patients have been randomized or the phase III investigational arm has been selected. The primary endpoint of investigator-assessed PFS will be evaluated based on RECIST 1.1 guidelines. Secondary endpoints include investigator-assessed ORR according to RECIST 1.1 guidelines, percentage of patients who initiate cycle 6 while receiving doublet chemotherapy, OS, and safety. This portion of the trial will also explore time to second-line therapy or death. Additional exploratory endpoints include enumeration of circulating tumor cells as a surrogate marker for efficacy, and correlative studies. Since the contributions of each drug to each doublet can, in principle, be deconvolved by comparison with the other arms, the design of the phase II portion presents certain opportunities for testing of putative predictive markers and for predictive marker discovery. Correlative sampling includes archival tumor tissue, which will be analyzed for molecular profiles, including protein, tumor cell mutations and gene expression. Plasma samples are also being obtained at baseline and cycle 3 in order to characterize tumor-derived cell-free DNA using digital polymerase chain reaction or whole-exome sequencing. Secreted protein acidic and rich in cysteine (SPARC) has been hypothesized to be a potential predictive biomarker for *nab*-paclitaxel efficacy, and higher SPARC expression has been reported in TNBC [35–37]. However, clinical data associating SPARC status with treatment efficacy in breast cancer are equivocal [35, 36, 38, 39]. Analysis of SPARC is not currently prioritized in the phase II portion of the trial.

Identification of the *nab*-paclitaxel experimental arm for use in the phase III portion of the trial will be based on an algorithm ranking the performance of five key efficacy and safety endpoint parameters preselected by the steering committee as well as the total phase II efficacy and safety data (Table 2). The 5 parameters include the hazard ratio (HR) of PFS (*nab*-paclitaxel plus gemcitabine/*nab*-P plus carboplatin), ratio of ORR, percentage of patients who initiated cycle 6 while receiving doublet combination therapy, percentage of patients who discontinued all study treatment due to adverse events (AEs), and percentage of patients with myelosuppression-related events. The *nab*-paclitaxel experimental arms from the phase II portion of the trial will be ranked by endpoint, and the *nab*-paclitaxel regimen with the more desirable treatment effect with respect to the evaluated endpoint will receive the higher rank. The *nab*-paclitaxel regimen with the highest total rank in the five endpoint parameters will be selected as the experimental arm in the phase III portion of the trial if the totality of the data supports the conclusion. The PFS and ORR efficacy endpoints each carry twice the weight of the remaining three endpoints. A *nab*-paclitaxel experimental arm should demonstrate greater efficacy in both PFS and ORR or in either PFS or ORR and at least two of the three remaining efficacy and/or safety endpoint parameters in order to be identified as the experimental arm for the phase III portion of the trial. This ranking algorithm will be used when approximately 144 total PFS events from 3 arms have been observed. The gemcitabine plus carboplatin regimen in the phase II portion of the study will not be ranked but will rather serve as a reference for descriptive comparisons between the two *nab*-paclitaxel experimental arms.

**Table 2:** Five parameters for selection of the *nab*-paclitaxel regimen for phase III evaluation

Efficacy and safety endpoint parameters
HR of PFS (*nab*-P + Gem/*nab*-P + Carbo)^a^
Ratio of ORR^a^
Percentage of patients who initiated cycle 6 while receiving doublet combination therapy
Percentage of patients with myelosuppression-related events^b^
Percentage of patients who discontinued all study treatment due to AEs
= Rank sum

^a^Carries twice the weight of the remaining three endpoints

^b^Percentage of patients with myelosuppression-related events is the percentage of patients with any of the following events: grade 3/4 neutropenia, grade 3/4 thrombocytopenia, grade 3/4 anemia, febrile neutropenia AEs, grade 3/4 bleeding AEs, red blood cell transfusion, or platelet transfusion. Each patient will be counted only once in the total percentage

*AE* adverse event, *Carbo* carboplatin, *Gem* gemcitabine, *HR* hazard ratio, *nab*-P *nab*-paclitaxel, *ORR* overall response rate, *PFS* progression-free survival

### Phase III portion of the study

The primary objective of the phase III portion of this trial is to compare the PFS of patients receiving the selected *nab*-paclitaxel regimen from the phase II portion of the trial (*nab*-paclitaxel plus either gemcitabine or carboplatin) with that of patients receiving gemcitabine plus carboplatin, as assessed by independent radiologist(s) blinded to treatment assignment using RECIST 1.1 guidelines. This portion will be an active comparator-controlled, randomized, multicenter, open-label study designed to compare treatment arm 1 (*nab*-paclitaxel combination treatment arm selected from the phase II portion of the study) with treatment arm 2 (gemcitabine 1000 mg/m^2^ plus carboplatin AUC 2 mg × min/mL), both administered IV on days 1 and 8 of a 21-day cycle. Approximately 550 patients will be randomized 1:1 (*n* = 275 patients per arm), stratified by DFI (≤1 year versus > 1 year) and prior adjuvant or neoadjuvant taxane treatment (yes or no), into the phase III portion of this study. The ORR, OS, investigator-assessed PFS, disease control rate, duration of response, and safety will also be evaluated. In addition, time to second-line therapy or death, healthcare resource utilization, quality of life, and analysis of tumor samples (based on phase II observations) in patients who agree to participate will be explored.

### Treatment duration and modifications

Treatment in both the phase II and III portions will continue until progressive disease or unacceptable toxicity. In the event of a hypersensitivity reaction or a clear toxicity to one of the treatment components, the investigator will permanently discontinue the suspect agent and continue monotherapy treatment with the remaining agent. If the toxicity is believed to be caused by both treatment components, both components will be modified or discontinued.

### Follow-up

All patients will be monitored for new or existing AEs for 28 days after treatment discontinuation using the NCI CTCAE Version 4.0. Follow-up will include physical examination and weight; vital signs; concomitant medication evaluation, including opioid analgesic consumption and new anticancer treatment; concomitant procedure evaluation; Eastern Cooperative Oncology Group performance status; AE evaluation; complete blood count with differential, including red blood cell count, hemoglobin, hematocrit, white blood cell count, absolute neutrophil count, and platelet count; and a chemistry panel.

Follow-up for survival will be monitored every 3 months after treatment discontinuation until a patient dies or the study closes. Information regarding the start of new anticancer treatment(s) and subsequent disease progression will be collected. This evaluation may be conducted by record review and/or telephone contact with the patient’s treating physician.

### Sample size and statistical methods

A sample size of 80 patients per arm was selected for the phase II portion of the study to permit qualitative comparisons of toxicity between the 2 *nab*-paclitaxel arms and to provide preliminary data on antitumor activity. This should also provide an approximate 80 % marginal power with a 2-sided significance level of 0.2 to differentiate an experimental regimen and the control arm, assuming a median PFS of 7.1 months for patients in the experimental arm and 4.6 months for those in the control arm. Exploratory pairwise comparisons will test the effect of *nab*-paclitaxel when 96 PFS events have been observed from 160 patients.

The phase III portion of the study is designed to enroll 550 patients (330 PFS events), which provides an approximate 90 % power to detect a HR of 0.70 for PFS with a 2-sided 5 % significance level. Assuming a median PFS of 4.6 months for the gemcitabine plus carboplatin arm, this represents a 43 % improvement in median PFS for the *nab*-paclitaxel arm.

### Randomization

Central randomization via a permuted-block design and an interactive voice response system will be implemented for both the phase II and III portions of the study.

### Data management and analysis

A data monitoring committee (DMC) will be established to review patient disposition, demographics, cancer history and baseline lesion status, AEs by grade classification, and myelosuppression by grade classification. For the phase II portion of the study, the DMC will review ongoing safety and dosing data when 20 patients are randomized per arm (total of 60 patients) and also when the last randomized patient from this initial group finishes the first treatment cycle. When approximately 100 PFS events are observed in the phase III portion of the study, the DMC may recommend stopping the study early for futility if the conditional power is ≤ 5 %.

### Study population definitions

The primary efficacy analysis will be performed on the intent-to-treat population, which includes all randomized patients regardless of whether they received any investigational product (IP) or had any efficacy assessments collected. Confirmatory efficacy analysis will be performed on the efficacy-evaluable population, which includes all randomized patients who meet the eligibility criteria, receive at least one dose of assigned IP, and have at least one baseline and post-baseline efficacy assessment(s). Safety and tolerability analyses will be performed on the treated population, which includes all randomized patients who receive at least one dose of IP.

## Discussion

An optimal treatment for TNBC has not yet been identified. A lack of a validated target for development of molecular-oriented therapy and the poor prognosis of patients with advanced-stage disease make TNBC a significant challenge for both patients and oncologists [2, 3]. Higher response rates observed with combination chemotherapy regimens may support the role of such treatments for the management of mTNBC [8]. In patients with TNBC, relapses occur frequently following a short DFI and accompanied by a high tumor burden coupled by symptomatic visceral involvement [1]. Whether this short DFI is indicative of primary chemoresistance remains to be determined. With respect to high tumor burden and symptomatic visceral involvement, rapid disease control is of paramount importance and may be best achieved with combination chemotherapy. However, combination treatments for patients with TNBC are difficult to explore in a landscape with no well-defined standard of care for comparison. The tnAcity trial will further current understanding by potentially identifying a suitable first-line chemotherapeutic combination regimen for use in future clinical trials, including those with targeted agents.

Chemotherapy currently remains the best systemic treatment strategy for patients with TNBC. Various molecular subtypes of TNBC have been identified [40], although at this time there is no single molecular alteration common to all TNBC tumors. Given this disease heterogeneity and the lack of a shared targetable mutation for drug therapy development, chemotherapy continues to be the foundation of effective treatment options, with a broad application that addresses the profound chemosensitivity of these tumors. Furthermore, some trials of targeted agents are restricted to specific aberrations and often address only a subset of patients [9, 16]. This appears to be true in the adjuvant setting as well, since the addition of bevacizumab to early-stage adjuvant chemotherapy did not improve outcomes [41]. In the metastatic disease setting, the poly ADP ribose polymerase-like agent iniparib added to a backbone of gemcitabine plus carboplatin did not significantly improve disease-free survival in a large phase III trial of patients with TNBC [9]. The importance of establishing an effective chemotherapy regimen for patients with TNBC is further emphasized by trials with targeted agents, since chemotherapy can serve as a backbone for testing these agents in selected patients with corresponding mutations. A recent meta-analysis of 12 trials comprising 2054 patients with TNBC found that, when coupled with conventional chemotherapy, targeted therapy demonstrated gains in the PFS of these patients [42]. Combining cytotoxic agents such as sb-paclitaxel, docetaxel, capecitabine, anthracyclines, gemcitabine, and vinorelbine with the targeted agent bevacizumab resulted in a superior PFS when compared with chemotherapy alone. Similar results for PFS were reported for treatment with gemcitabine or capecitabine plus sorafenib versus chemotherapy alone in the second-line setting.

The tnAcity trial will use a ranking algorithm based on predefined efficacy and safety parameters to systematically select the most clinically beneficial *nab*-paclitaxel-based regimen from the phase II portion of the study for comparison with the control arm in the phase III portion (Table 2). In this algorithm, the PFS and ORR endpoints carry twice the weight of the remaining three endpoints to reflect the importance of efficacy in this evaluation. The pick-the-winner algorithm balances the importance of efficacy with the need to minimize toxicities, especially those that limit the ability to deliver active therapy. The *nab*-paclitaxel combination doses and schedules evaluated in this study were chosen carefully based on the experience with *nab*-paclitaxel/gemcitabine in pancreatic cancer and *nab*-paclitaxel/carboplatin in NSCLC, while matching the doses and schedule of the gemcitabine/carboplatin regimen evaluated in the TNBC phase II and III trials of iniparib and commonly used in patients with TNBC [9, 16, 32, 33, 37, 43]. When compared with the dose used to treat pancreatic cancer, *nab*-paclitaxel/gemcitabine dose intensity was reduced in the tnAcity trial to decrease associated neurotoxicity and myelosuppression. The *nab*-paclitaxel/carboplatin treatment regimen was also slightly modified compared with the schedule for NSCLC, with the introduction of a 1-week treatment break to reduce treatment-related AEs. Of note, the recently fully recruited neoadjuvant ADAPT trial (WSG; West German Study Group) has used the same 2 *nab-*paclitaxel chemotherapy combinations as the phase II part of tnAcity for 12 weeks before surgery [44]. Final pCR results are expected for the end of 2015 and may substantiate the results of the ranking algorithm.

The tnAcity trial will enable robust treatment analysis of a prospectively defined patient population in mTNBC. The trial results may identify a *nab*-paclitaxel-based cytotoxic chemotherapy combination as a new first-line standard treatment regimen for mTNBC. Additionally, biomarker analyses may advance current understanding of TNBC biology and response to treatment.

## Trial status

The trial is currently enrolling patients.

## Additional file

Additional file 1:
**Approving ethical bodies. This file lists name, type, and location of ethical bodies that approved this study.** (XLSX 22 kb)

## Abbreviations

AE: adverse event
AUC: area under the curve
*BRCA*: breast cancer susceptibility gene
DFI: disease-free interval
DMC: data monitoring committee
ER: estrogen receptor
HER2: human epidermal growth factor receptor 2
HR: hazard ratio
IP: investigational product
IV: intravenous
mTNBC: metastatic triple-negative breast cancer
*nab*-P: *nab*-paclitaxel
NSCLC: non-small cell lung cancer
ORR: overall response rate
OS: overall survival
pCR: pathological complete response
PFS: progression-free survival
PR: progesterone receptor
q2w: every 2 weeks
q3w: every 3 weeks
qw 3/4: the first 3 of 4 weeks
RECIST: Response Evaluation Criteria In Solid Tumors
sb-: solvent-based
SPARC: secreted protein acidic and rich in cysteine
tnAcity: Triple-Negative Albumin-bound paclitaxel Combination International Treatment Study
TNBC: triple-negative breast cancer.

## References

[1] Liedtke C, Mazouni C, Hess KR, Andre F, Tordai A, Mejia JA (2008). Response to neoadjuvant therapy and long-term survival in patients with triple-negative breast cancer. J Clin Oncol.

[2] Hudis CA, Gianni L (2011). Triple-negative breast cancer: an unmet medical need. Oncologist.

[3] Bauer KR, Brown M, Cress RD, Parise CA, Caggiano V (2007). Descriptive analysis of estrogen receptor (ER)-negative, progesterone receptor (PR)-negative, and HER2-negative invasive breast cancer, the so-called triple-negative phenotype: a population-based study from the California Cancer Registry. Cancer.

[4] Dent R, Trudeau M, Pritchard KI, Hanna WM, Kahn HK, Sawka CA (2007). Triple-negative breast cancer: clinical features and patterns of recurrence. Clin Cancer Res.

[5] Shastry M, Yardley DA (2013). Updates in the treatment of basal/triple-negative breast cancer. Curr Opin Obstet Gynecol.

[6] Lin NU, Claus E, Sohl J, Razzak AR, Arnaout A, Winer EP (2008). Sites of distant recurrence and clinical outcomes in patients with metastatic triple-negative breast cancer: high incidence of central nervous system metastases. Cancer.

[7] Rouzier R, Perou CM, Symmans WF, Ibrahim N, Cristofanilli M, Anderson K (2005). Breast cancer molecular subtypes respond differently to preoperative chemotherapy. Clin Cancer Res.

[8] National Comprehensive Cancer Network (2015). Clinical practice guidelines in oncology: breast cancer.

[9] O’Shaughnessy J, Schwartzberg L, Danso MA, Miller KD, Rugo HS, Neubauer M (2014). Phase III study of iniparib plus gemcitabine and carboplatin versus gemcitabine and carboplatin in patients with metastatic triple-negative breast cancer. J Clin Oncol.

[10] Partridge AH, Rumble RB, Carey LA, Come SE, Davidson NE, Di Leo A (2014). Chemotherapy and targeted therapy for women with human epidermal growth factor receptor 2-negative (or unknown) advanced breast cancer: American Society of Clinical Oncology Clinical Practice Guideline. J Clin Oncol.

[11] Cardoso F, Costa A, Norton L, Senkus E, Aapro M, Andre F (2014). ESO-ESMO 2nd international consensus guidelines for advanced breast cancer (ABC2)^†^. Ann Oncol.

[12] Harbeck N, Marschner N, Untch M, Decker T, Hegewisch-Becker S, Jackisch C (2014). Second International Consensus Conference on Advanced Breast Cancer (ABC2), Lisbon, 11/09/2013: The German Perspective. Breast Care (Basel).

[13] Fan Y, Xu BH, Yuan P, Ma F, Wang JY, Ding XY (2013). Docetaxel-cisplatin might be superior to docetaxel-capecitabine in the first-line treatment of metastatic triple-negative breast cancer. Ann Oncol.

[14] Hu XC, Zhang J, Xu BH, Cai L, Ragaz J, Wang ZH (2015). Cisplatin plus gemcitabine versus paclitaxel plus gemcitabine as first-line therapy for metastatic triple-negative breast cancer (CBCSG006): a randomised, open-label, multicentre, phase 3 trial. Lancet Oncol.

[15] Gucalp A, Traina TA (2011). Triple-negative breast cancer: adjuvant therapeutic options. Chemother Res Pract.

[16] O’Shaughnessy J, Osborne C, Pippen JE, Yoffe M, Patt D, Rocha C (2011). Iniparib plus chemotherapy in metastatic triple-negative breast cancer. N Engl J Med.

[17] Isakoff SJ, Mayer EL, He L, Traina TA, Carey LA, Krag KJ (2015). TBCRC009: a multicenter phase II clinical trial of platinum monotherapy with biomarker assessment in metastatic triple-negative breast cancer. J Clin Oncol.

[18] Tommiska J, Bartkova J, Heinonen M, Hautala L, Kilpivaara O, Eerola H (2008). The DNA damage signalling kinase ATM is aberrantly reduced or lost in BRCA1/BRCA2-deficient and ER/PR/ERBB2-triple-negative breast cancer. Oncogene.

[19] Tassone P, Di Martino MT, Ventura M, Pietragalla A, Cucinotto I, Calimeri T (2009). Loss of BRCA1 function increases the antitumor activity of cisplatin against human breast cancer xenografts in vivo. Cancer Biol Ther.

[20] Tutt A, Ellis P, Kilburn L, Gilett C, Pinder S, Abraham J (2014). TNT: a randomized phase III trial of carboplatin (C) compared with docetaxel (D) for patients with metastatic or recurrent locally advanced triple negative or BRCA1/2 breast cancer (CRUK/07/012).

[21] Rugo HS, Olopade O, DeMichel A, van’t Veer L, Buxton M, Hylton N (2013). Veliparib/carboplatin plus standard neoadjuvant therapy for high-risk breast cancer: first efficacy results from the I-SPY 2 TRIAL.

[22] Sikov WM, Berry DA, Perou CM, Singh B, Cirrincione CT, Tolaney SM (2015). Impact of the addition of carboplatin and/or bevacizumab to neoadjuvant once-per-week paclitaxel followed by dose-dense doxorubicin and cyclophosphamide on pathologic complete response rates in stage II to III triple-negative breast cancer: CALGB 40603 (Alliance). J Clin Oncol.

[23] Roy V, LaPlant BR, Gross GG, Bane CL, Palmieri FM (2009). North Central Cancer Treatment Group. Phase II trial of weekly nab (nanoparticle albumin-bound)-paclitaxel (nab-paclitaxel) (Abraxane) in combination with gemcitabine in patients with metastatic breast cancer (N0531). Ann Oncol.

[24] Lobo C, Lopes G, Baez O, Castrellon A, Ferrell A, Higgins C (2010). Final results of a phase II study of nab-paclitaxel, bevacizumab, and gemcitabine as first-line therapy for patients with HER2-negative metastatic breast cancer. Breast Cancer Res Treat.

[25] Hamilton E, Kimmick G, Hopkins J, Marcom PK, Rocha G, Welch R (2013). Nab-paclitaxel/bevacizumab/carboplatin chemotherapy in first-line triple negative metastatic breast cancer. Clin Breast Cancer.

[26] Gradishar WJ, Tjulandin S, Davidson N, Shaw H, Desai N, Bhar P (2005). Phase III trial of nanoparticle albumin-bound paclitaxel compared with polyethylated castor oil-based paclitaxel in women with breast cancer. J Clin Oncol.

[27] Gradishar WJ, Krasnojon D, Cheporov S, Makhson AN, Manikhas GM, Clawson A (2009). Significantly longer progression-free survival with nab-paclitaxel compared with docetaxel as first-line therapy for metastatic breast cancer. J Clin Oncol.

[28] Gradishar WJ, Krasnojon D, Cheporov S, Makhson AN, Manikhas GM, Clawson A (2012). Phase II trial of nab-paclitaxel compared with docetaxel as first-line chemotherapy in patients with metastatic breast cancer: final analysis of overall survival. Clin Breast Cancer.

[29] Abraxane [package insert]. Summit, NJ; Celgene Corporation. 2015.

[30] Untch M, Jackisch C, Schneeweiss A, Conrad B, Aktas B, Kenkert C (2014). A randomized phase III trial comparing neoadjuvant chemotherapy with weekly nanoparticle-based paclitaxel with solvent-based paclitaxel followed by anthracyline/cyclophosphamide for patients with early breast cancer (GeparSepto); GBG 69.

[31] Clinicaltrials.gov. http://www.clinicaltrials.gov/ct2/results?term=nct01207102&Search=Search. Accessed 14 October 2015.

[32] Socinski MA, Bondarenko I, Karaseva NA, Makhson AM, Vynnychenko I, Okamoto I (2012). Weekly nab-paclitaxel in combination with carboplatin versus solvent-based paclitaxel plus carboplatin as first-line therapy in patients with advanced non-small-cell lung cancer: final results of a phase III trial. J Clin Oncol.

[33] Von Hoff DD, Ervin T, Arena FP, Chiorean EG, Infante J, Moore M (2013). Increased survival in pancreatic cancer with nab-paclitaxel plus gemcitabine. N Engl J Med.

[34] National Comprehensive Cancer Network (2015). Clinical practice guidelines in oncology: pancreatic adenocarcinoma.

[35] Yardley DA, Daniel B, Inhorn RC, Vazquez ER, Trieu V, Motamed K (2010). SPARC microenvironment signature (SMS) analysis of a phase II trial of neoadjuvant gemcitabine (G), epirubicin (E), and *nab*-paclitaxel (*nab*-P) in locally advanced breast cancer (LABC).

[36] Yardley DA, Raefsky E, Castillo R, Lahiry A, LoCicero R, Thompson D (2009). Results of a multicenter pilot study of weekly nab paclitaxel, carboplatin with bevacizumab and trastuzumab as neoadjuvant therapy in HER2+ locally advanced breast cancer with SPARC correlatives.

[37] Von Hoff DD, Ramanathan RK, Borad MJ, Laheru DA, Smith LS, Wood TE (2011). Gemcitabine plus nab-paclitaxel is an active regimen in patients with advanced pancreatic cancer: a phase I/II trial. J Clin Oncol.

[38] Blackwell KL, Hamilton EP, Rocha G, Gainey M, Trieu VN, Motamed K (2010). SPARC microenvironment signature (SMS) in patients treated with nab-paclitaxel(nabP)/carboplatin (C)/bevacizumab(B) for triple-negative metastatic breast cancer (TNMBC).

[39] Lindner JL, Loibl S, Denkert C, Ataseven B, Fasching PA, Pfitzner BM (2015). Expression of secreted protein acidic and rich in cysteine (SPARC) in breast cancer and response to neoadjuvant chemotherapy. Ann Oncol.

[40] Lehmann BD, Bauer JA, Chen X, Sanders ME, Chakravarthy AB, Shyr Y (2011). Identification of human triple-negative breast cancer subtypes and preclinical models for selection of targeted therapies. J Clin Invest.

[41] Cameron D, Brown J, Dent R, Jackisch C, Mackey J, Pivot X (2013). Adjuvant bevacizumab-containing therapy in triple-negative breast cancer (BEATRICE): primary results of a randomised, phase 3 trial. Lancet Oncol.

[42] Clark O, Botrel TE, Paladini L, Ferreira MB (2014). Targeted therapy in triple-negative metastatic breast cancer: a systematic review and meta-analysis. Core Evid.

[43] Socinski MA, Manikhas GM, Stroyakovsky DL, Makhson AN, Cheporov SV, Orlov SV (2010). A dose finding study of weekly and every-3-week nab-Paclitaxel followed by carboplatin as first-line therapy in patients with advanced non-small cell lung cancer. J Thorac Oncol.

[44] Hofmann D, Nitz U, Gluz O, Kates RE, Schinkoethe T, Staib P (2013). WSG ADAPT – adjuvant dynamic marker-adjusted personalized therapy trial optimizing risk assessment and therapy response prediction in early breast cancer: study protocol for a prospective, multi-center, controlled, non-blinded, randomized, investigator initiated phase II/III trial. Trials.

