# Erratum to: ‘Phase II/III weekly *nab*-paclitaxel plus gemcitabine or carboplatin versus gemcitabine/carboplatin as first-line treatment of patients with metastatic triple-negative breast cancer (the tnAcity study): study protocol for a randomized controlled trial

**DOI:** 10.1186/s13063-016-1195-6

**Published:** 2016-02-03

**Authors:** Denise A. Yardley, Adam Brufsky, Robert E. Coleman, Pierfranco F. Conte, Javier Cortes, Stefan Glück, Jean-Mark A. Nabholtz, Joyce O’Shaughnessy, Robert M. Beck, Amy Ko, Markus F. Renschler, Debora Barton, Nadia Harbeck

**Affiliations:** Sarah Cannon Research Institute and the Tennessee Oncology, PLLC, 250 25th Avenue North, Suite 100, Nashville, TN 37203 USA; University of Pittsburgh Medical Center, Pittsburgh, PA USA; Weston Park Hospital, Sheffield Cancer Research Center, Sheffield, England; Department of Surgery, Oncology and Gastroenterology, University of Padova, Padova, and Istituto Oncologico Veneto IRCCS, Padova, Italy; Vall d’Hebron Institute of Oncology (VHIO), Barcelona, Spain; Celgene Corporation, Summit, NJ USA; Centre de Lutte Contre le Cancer d’Auvergne, Clermont Ferrand, France; Texas Oncology-Baylor Charles A. Sammons Center; US Oncology, Dallas, TX USA; Breast Center, University of Munich, Munich, Germany

Unfortunately, the original version of this article [1] contained a few formatting errors which have been corrected via an update. In the eighth paragraph of the background there was an error. The original read as, “A phase II study evaluating the combination of nab-paclitaxel plus carboplatin as 199 first-line treatment for mTNBC is currently under way [31].” But should have read as “A phase II study evaluated the combination of nab-paclitaxel plus carboplatin a first-line treatment for mTNBC [31].”
